# A Professional Specimen. Sayles vs. Rumble

**Published:** 1855-07

**Authors:** 


### ARTICLE XV.

### *A Professional Specimen. Sayles* vs. *Rumble.*

THIS was an action for slander, imputing to the plaintiff, a dentist, that he mixed farthings with the gold used by him in making false teeth. The defendant pleaded "Not Guilty."

Mr. Mellor, Q. C., and Mr. W. H. Adams appeared for the plaintiff; and Mr. Macauley, Q. C., and Mr. Hayes for the defendant.

The principal witness was Mary Ann Metcalf, the chambermaid at the White Hart, who went to consult the defendant, a chemist and druggist in Lincoln, about her teeth; and acknowledged, amid much laughter, that she wanted five false teeth. Some 12 years before she had visited Mr. Sayles for a similar purpose, and then had one false tooth, which she had worn ever since. This was shown to Mr. Rumble, who said that it was not such good gold as it ought to be; and that he himself used only the best; that he gave 4 guineas for his gold, but that Mr. Sayles melted down farthings with sovereigns. He agreed to supply Miss Metcalf at the rate of 15s. per tooth; but when they were made, she found that they would not fit her, and declined paying for them. Mr. Rumble sued her in the County Court; and the report of that trial in the *Lincolnshire Chronicle* brought to the plaintiff's knowledge the slanderous words of which he complained.

Mr. Sayles upon cross-examination stated that he had formerly been a goldsmith and silversmith, and that he bound all his apprentices not to practice within 20 miles of Lincoln. He used silver as an alloy, and never copper; but he had a peculiar method of mixing his gold, which was a secret.

Mr. Macauley, in addressing the jury for the defendant, ridiculed the notion that the plaintiff had sustained any injury from the words spoken by the defendant to the chambermaid of the White Hart while he was operating upon her teeth, and suggested to the jury the propriety of giving to him one of those coins the mention of which had so much offended him, but of which he must have been very short when he thought of bringing this action.

The learned Judge summed up, and they found a verdict for the plaintiff—Damages, one farthing.

